# Reversibility of Neuropsychiatric Adverse Events after Switching to Darunavir/Cobicistat or Doravirine in Men on INSTI-Based Regimen

**DOI:** 10.3390/v16071083

**Published:** 2024-07-05

**Authors:** José Antonio Mata-Marín, Carina Aurora Juárez-Contreras, Mara Soraya Rodríguez-Evaristo, Olivia Concepción Martínez-Carrizales, Ericka Pompa-Mera, Alberto Chaparro Sánchez, Salma Triana-González, Ana Luz Cano-Díaz, Jesús Enrique Gaytán-Martínez

**Affiliations:** 1 Infectious Diseases Department, Hospital de Infectología, “La Raza” National Medical Center, Instituto Mexicano del Seguro Social, Mexico City 02990, Mexico; jamatamarin@gmail.com (J.A.M.-M.);; 2 Internal Medicine Department, Hospital de Especialidades, “La Raza” National Medical Center, Instituto Mexicano del Seguro Social, Mexico City 02990, Mexico; 3 Departamento de Posgrados, Escuela Superior de Medicina, Instituto Politécnico Nacional, Mexico City 07360, Mexico; 4 Psychiatric Department, Hospital de Infectología, “La Raza” National Medical Center, Instituto Mexicano del Seguro Social, Mexico City 02990, Mexico; 5 Research Unit, Hospital de Infectología, “La Raza” National Medical Center, Instituto Mexicano del Seguro Social, Mexico City 02990, Mexico; erickanelly@yahoo.com.mx

**Keywords:** integrase, neuropsychiatric, darunavir, doravirine, insomnia

## Abstract

Integrase strand transfer inhibitors (INSTI) are associated with neuropsychiatric adverse events (NPAEs). The aim of this study was to evaluate improvements in NPAEs after switching an INSTI-based regimen to darunavir/cobicistat (DRV/c) or doravirine (DOR). **Methods**: A prospective cohort study was conducted to evaluate the reversibility of NPAEs via the Patient Health Questionnaire (PHQ-9), the Insomnia Severity Index (ISI), and the Hospital Anxiety and Depression Scale (HADS-A and D) in patients who started antiretroviral therapy with dolutegravir (DTG) or bictegravir (BIC). These patients were switched to DRV/c or DOR. Scales were compared at the moment of the switch and 12 weeks later. **Results**: We included 1153 treatment-naïve men, 676 (58.7%) with BIC and 477 (41.3%) with DTG. A total of 32 (2.7%) experienced NPAEs that led to discontinuation. Insomnia was found in 20 patients; depression via PHQ-9 in 21 patients, via HADS-D in 5 patients, and anxiety via HADS-A in 12 patients. All of them were evaluated by a psychiatrist at the moment of the symptoms; 7 (21.8%) started psychotropic drugs. After 12 weeks of follow-up, PHQ-9, ISI, HADS-A, and HADS-D decreased, with a *p*-value ≤ 0.05. **Conclusions**: NPAEs seem to improve after switching to a DRV/c- or DOR-based regimen after the first 4 and 12 weeks.

## 1. Introduction

Neuropsychiatric adverse events (NPAEs) such as depression, anxiety, and sleep disorders are common in persons living with HIV (PLWH) and are associated with worse health-related quality of life outcomes, social phobia, living arrangement, sexual dysfunction, and poor antiretroviral therapy (ART) adherence [1,2]. In addition, high prevalence of sleep disorders in PWH have close association with psychiatric disorders such as depression and anxiety, as well as increased cardiovascular risk and obesity, which are frequently undiagnosed [3]. In recent years, data from real-world studies have raised concerns about the safety of integrase strand transfer inhibitors (INSTI) in real-life settings [4]. In a large cohort, the rate of discontinuation of dolutegravir (DTG) because of NPAEs was significantly higher than for other INSTIs (at almost 6% within 12 months); and almost three-fold-higher discontinuation rates were observed amongst women and older patients [5]. In another cohort of PWH who initiated a fixed-dose combination of bictegravir/emtricitabine/tenofovir alafenamide (BIC/F/TAF) in routine clinical practice, the estimated rates of discontinuation due to any AE or NPAEs during the first 6 months of exposure were 5.3% and 3.3%, respectively, and NPAEs were mild to moderate and quickly resolved after discontinuation [6].

While immediate initiation of antiretroviral therapy was proposed by the WHO; the early diagnosis and evaluation of mental-health- and psychiatric-related illnesses in PWH is also important since its influence on ART adherence, quality of life, morbidity, and mortality rates are well known [7]. The Hospital Anxiety and Depression Scale (HADS) provides healthcare professionals with a highly effective screening tool for clinical depression in PWH [8]. The Patient Healthcare Questionnaire 9 (PHQ-9) has also shown reasonable accuracy in classifying cases of depression in primary care settings [9]. Sleep disorders have been evaluated in PWH via the Insomnia Severity Index (ISI), a 7-item instrument measuring the severity of insomnia, as well as via the Pittsburgh Sleep Quality Index (PSQI), a 19-item tool measuring overall sleep quality across seven domains [10,11].

Some studies support the idea that switching the INSTI-based regimen to a regimen with a lower neurotoxic profile would improve symptoms in PWH who experience insomnia, mood disorders, or neuropsychiatric symptoms [12,13]. The aim of this study was to evaluate improvements in NPAEs after switching an INSTI-based regimen to darunavir/cobicistat or doravirine.

## 2. Materials and Methods

A prospective cohort study was conducted from March 2021 to October 2023 in patients treated at Hospital de Infectología “La Raza” National Medical Center, Mexico City. This center is a third-level reference unit for the management of infectious diseases in people with social security coverage, and it is one of the biggest HIV clinics in the country. The follow-up period for the subjects was at initial evaluation before starting ART and then at 4 and 12 weeks after switching ART. The study was approved by the Local Committee for Health Research 3502 at the Hospital de Infectología, with the registration number R-2022-3502-152. Written informed consent was obtained from all participants before they answered a questionnaire, and their clinical data were collected in case report form.

### 2.1. Study Population

Individuals eligible for screening included male PWH ≥ 18 years old without major neuropsychiatric comorbidities (major depression with psychotic symptoms or suicidal ideation, drug abuse/dependence, dementia, or psychosis) receiving BIC/FTC/TAF or DTG/3TC/ABC. We include in this study those with grade-3 NPAEs who were switched to DRV/c plus tenofovir/emtricitabine (DRV/c plus TFD/FTC) or doravirine/lamivudine/tenofovir (DOR/3TC/TDF) and complete the follow-up at 4 and 12 weeks after switch.

### 2.2. Measurements

This study included visits at baseline and weeks 4 and 12 for participants who were switched for NPAEs. NPAEs, such as depression, anxiety, and sleep quality, were assessed using 4 standardized, validated self-reported scales: the Hospital Anxiety and Depression scale (HADS), which provide healthcare professionals with a highly effective screening tool for clinical depression and anxiety in PWH [8]; the Patient Healthcare Questionnaire 9 (PHQ-9), a tool with reasonable accuracy in classifying cases of depression in primary care settings [9]; the Insomnia Severity Index (ISI), a 7-item instrument measuring the severity of insomnia [10]; and the Pittsburgh Sleep Quality Index (PSQI), a 19-item tool measuring overall sleep quality across 7 domains [11]. These questionnaires have been validated for their use in PWH and can be widely used for assessing sleep and mood disturbances. Participants completed the 4 questionnaires at each visit. If the patients had HADS-A ≥ 11 or HADS-D ≥ 11 or ISI ≥ 15 or PHQ-9 ≥ 15 or PSQI ≥ 8, associated with adverse events grade ≥ 3 or suicidality, they were switched to DRV/c + TFD/FTC or DOR/FTC/TDF. Patients’ adherence to a study-drug regimen was assessed by self-report.

### 2.3. Statistical Analysis

The qualitative data were analyzed through frequency and percentage tables. Quantitative measures, according to their non-normal distribution (Kolmogorov–Smirnov test), were summarized using median and interquartile ranges (IQR). We evaluate changes in questionnaires scores through the Wilcoxon signed-rank test; a *p* value ≤ 0.05 was considered statistically significant. All analyses were conducted using SPSS software (version 26; IBM^®^ SPSS^®^ Corp., Armonk, NJ, USA).

## 3. Results

A total of 1164 treatment-naïve male PWH were included after starting ART; of them, 11 participants (0.9%) were loss to follow-up, and 1153 who started antiretroviral therapy with the INSTI-based regimen were included for the analysis: 676 (58.7%) with BIC-based regimen and 477 (41.3%) with DTG-based regimen. The median age of the participants was 27 years (IQR 24-32), with a median CD4+ cell count of 253 cells (IQR 164-362) and an HIV-1 RNA viral load of 33,411 (IQR 9201-111205). Twenty (1.7%) patients had hypertension and four (0.3%) had diabetes type 2.

Regarding insomnia evaluated via ISI, 39 participants (3.4%) experienced sleep disturbances and insomnia (ISI ≥ 15 points and/or PSQI ≥ 8 points); they were recommended to take the drug in the morning; and in 19 (48%), insomnia and sleep disturbances resolved after 4 weeks.

After follow-up during the first 12 weeks, 32 participants (2.7%) experienced NPAEs that lead to discontinuation (the median time for discontinuation was 7 weeks (IQR 5–11)), 20 (62.5%) from BIC and 12 (37.5%) from DTG; there was no difference in discontinuation rate between BIC-based regimen with 20/676 (2.9%) and DTG-based regimen with 12/477 (2.5%). Adherence was high, with a median of 97% (IQR 95–100%). Insomnia via ISI was found in 20 patients (62.5%); ISI median was 19 (IQR 17–21). The median of PSQI was 14 points (IQR 12–15). Depression was found via PHQ-9 in 21 patients (65.6%), with median 19 points (IQR 15–22); via HADS-D in 5 patients (15.6%), with a median of 9 points (IQR 7–12); and anxiety via HADS-A in 12 patients (37.5%), with a median of 12 points (IQR 11–14) (Table 1).

After the evaluation by a psychiatrist at the moment of the symptoms, seven (21.8%), started psychotropic drugs, and three (42%) of them started two drugs; the most common were clonazepam in four (57%), citalopram in thee (42%), fluoxetine in three (42%), quetiapine in two (28%), and paroxetine in two (28%).

Some patients had more than one NPAE, which led to discontinuation; of them, 20 (62.5%) and 12 (37.5%) were switched to DRV/c plus TFV/FTC regimen and DOR/3TC/TDF, respectively.

After 12 weeks of follow-up after switch, PHQ-9 was 5 points (IQR 2–12) (*p* = 0.004); ISI was 7 points (IQR 4–12) (*p* = 0.001); HADS-A was 7 points (IQR 6–9) (*p* = 0.017); HADS D 8 (IQR 3–10) (*p* = 0.001); and PSQI 6 (6–7) (*p* = 0.035) (Table 2). All of the participants remained with viral load <40 copies/mm in the follow-up.

## 4. Discussion

In this prospective cohort, improvement of NPAEs measured with HADS-D, HADS-A, ISI, and PHQ-9 after switching a second-generation INSTI-based regimen to a DRV/c- or DOR-based regimen in PWH was found. We observed a significant reduction in anxiety, depression, and insomnia after 4 and 12 weeks of follow-up. We also identified significant improvements in subjective quality of sleep via PSQI score and self-reported improvement in daily activities.

We observed through objective measurements that when the switch was made to a DRV/c- or DOR-based regimen, not only do the questionnaire scores decrease, the self-reports of neuropsychiatric symptomatology also decrease.

Several studies showed that NPSAEs are associated with second-generation INSTI [4,5,6], and it is challenging for the clinician to decide the best choice for the patients with respect to starting a psychotropic drug or switching to an antiretroviral regimen.

The onset of antidepressant effects has great clinical relevance. Patients and those treating them need to know when to expect the timing of onset of clinical improvement with the selective serotonin reuptake inhibitors (SSRIs); resolution of symptoms with fluoxetine, sertraline, paroxetine, and citalopram will not be immediately perceived by patients, and this might impact quality of life and adherence to medical care [14]. Some studies have suggested that for currently available antidepressants, the average time for onset of antidepressant action is 13 days; but when considering full response criteria, this period goes up to 20 days [15]. In our health system, the most frequently prescribed antidepressant is sertraline, and it usually takes 4 to 6 weeks to fully work [16]; fluoxetine will start action by week 2, with over 75% starting to respond by week 4 [17]. In addition, side effects of antidepressant drugs, such as feeling sick, headaches, and trouble sleeping, are common. All of this implies that we must not limit clinical intervention in PWH with adverse effects to just add psychotropic drugs. Therefore, identifying other effective strategies might improve the quality of life of patients, as we found in this cohort.

If the decision is switching ART, we have two situations to consider: the first one is if the NPAEs will be reversible with another antiretroviral drug; and the second is how much time this new drug will taker to reverse the NPAEs. Some studies have demonstrated that when an INSTI-based regimen is switching, we could have reversibility of NPAEs, but improvement time has not been discussed [12,13]. Cabello-Ubeda et al. showed that switching DTG/3TC/ABC to DRV/c/FTC/TAF in PWH describing poor quality of sleep without insomnia complaints was associated with better sleep quality. Specifically, they observed a significative reduction in moderate-to-severe symptoms in the following sleep components: subjective quality of sleep, sleep latency, sleep duration, habitual sleep efficiency, and daytime dysfunction, similar to findings in this study regarding improvement in insomnia, anxiety, and depression [12].

If we consider all the patients who experience grade-3 NPAEs, they come to 51 (4.4%), but 19 (48%) of them who experience insomnia and sleep disturbances could continue with the INSTI-base regimen simply by changing the pill to a morning dose, as was described by Capetti et al. [18].

The effect of ART on sleep disturbances has been controversial for many reasons. Indeed, receiving an HIV diagnosis can represent an emotional experience, which can result in mood disorders, such as anxiety depression, and contribute to the development of sleep disturbances or worsen sleep quality [19]. On the other hand, the ART drugs class can be an important factor in developing sleep abnormalities. ART regimens based on efavirenz were found to have a significant association with sleep disturbances [20]. Unlike the discontinuation of another ART regimens, such as efavirenz, emtricitabine, and tenofovir, the therapy switch did not reverse sleep abnormalities [21]. We found a significant association between insomnia severity and improvement in sleep quality after switching to DRV/c plus TFD/FTC or DOR/3TC/TDF.

Interestingly, sleep disturbances in PWH are associated with impairments in immune function, low CD4+ cell counts, detectable HIV-1 RNA viral load, and limited physical activity [22]. In fact, derangements in lymphocyte count may contribute to HIV-associated inflammation and chronic immune activation [23]. One study by Bruno et al. found poor sleep quality (PSQI score > 5) in subjects with a higher body mass index (BMI) [24]. In this regard, INSTI such as BIC or DTG have been associated with excessive weight gain/obesity in a minority of PWH [25]. If the weight gain associated with the use of INSTI impacts sleep disturbances, it deserves more research in the future. Given the bidirectional association between sleep and depression, the multidisciplinary clinical approach must be based on the management of one, which may improve the other. Thus, the treatment of depression might help to improve sleep quality, reducing anxiety and depression in PWH [26].

Taramasso et al., in an observational and prospective study cohort, found a higher incidence of NPAEs in DTG compared to non-DTG-treated PWH, and most NPAEs resolve after an ART switch to a different drug, even one of the same family [13]. Moderate-to-severe NPAEs were not so common in this study; only 32 (2.7%) experienced NPAEs that lead to discontinuation, in contrast with other studies that found an incidence between 3.3% to 5.6% [13,19].

Regarding NPAEs on PWH in an INSTI regimen, women and older people had higher discontinuation rates compared with young men living with HIV [27]. Historically, it has been considered that women are 1.4–2 times more likely to report sleep disturbances, particularly insomnia, compared with men, as well as to develop chronic sleep problems, which could be due to higher psychosocial distress or emotional reactivity [28]. In the case of our study, since our HIV clinic only treats men living with HIV, we were not able to evaluate this association. Compared with the younger generation, older adults with HIV/AIDS may have a higher risk of mental health problems such as depression as they may experience more HIV-driven psychological challenges due to social stigma and discrimination. A study conducted in Ethiopia found that increased age was positively associated with depression, potentially due to a higher prevalence of chronic illnesses such as diabetes, hypertension, and heart disease, which have been theorized to be important predictors of depressive symptoms [29]. In this cohort, the median age in PWH who discontinued ART due to NPAEs was 25 (23–29), and only 25 patients had chronic illnesses, so we cannot thoroughly evaluate this relationship between age and depression.

At the moment, second-generation INSTI are not contraindicated in patients with subjacent neuropsychiatric disease, but some guidelines recommend that INSTI-containing regimens should be used with caution in patients with a pre-existing history of any psychiatric illness, including depression [21,30]. Patients with NPAEs associated with ART, such as sleep disturbances, could increase their risk of cardiovascular disease, obesity, and mood disorders [3,31], affecting their quality of life and intensifying their symptoms of depression and anxiety [22], and side effects could predict low adherence [23].

This study has important limitations. First, it is an observational study without a control group to establish if, in the next weeks, even without switching ART, neuropsychiatric scales might improve because of patient adaptation or comedications. Second, the study had an open-label design. Third, the short follow-up period; longer follow-up is needed to observe if greater NPAEs occur over time. Fourth, we were unable to measure markers in plasma or cerebrospinal fluid associated with central nervous system damage as we do not have these resources at our laboratory.

Despite these limitations, this study has some strengths; for example, every patient was objectively evaluated with validated self-applied questionnaires, and none of them had insomnia, anxiety, depression, or suicidality before starting ART; further, patients were followed-up prospectively with the same questionnaires at each visit and were evaluated by a psychiatrist who initiated psychotropic drugs according to assessment.

The findings of this study support the recommendation of an early switch in ART regimen when NPAEs are identified, which ultimately improves the adherence and outcomes of people living with HIV.

A clinical trial with a control group maintaining the INSTI-based regimen and a longer period of follow-up are necessary in order to acquire a better understanding of whether time could improve the NPAEs of these patients.

## Figures and Tables

**Table 1:** Characteristics of the study population (*n* = 1153).

Parameter	PWH Who Continued with INSTI. *n* = 1121	PWH with NPAES That Lead to Discontinuation *n* = 32
Age, median (IQR), years	27 (24–32)	25 (22–28)
CD4 +, median (IQR) cells/mm^3^	253 (164–362)	237 (168–372)
HIV viral load, median (IQR), log10	4.4 (3.9–4.9)	4.4 (3.9–4.8)
BIC-based regimen, *n* (%)	676 (58.7%)	20 (62.5%)
DTG-based regimen, *n* (%)	477 (41.3%)	12 (37.5%)
NPAES that lead to discontinuation, *n* (%)	32 (2.7%)	
PHQ-9, median (IQR), points	4 (1–7)	19 (15–22)
ISI, median, (IQR), points	5 (1–9)	19 (17–21)
HADS-A, median, (IQR), points	2 (1–5)	12 (11–194)
HADS-D, (IQR), median, points	2 (1–5)	9 (7–12)
PSQI median, (IQR) points	4 (1–7)	14 (12–15)
Switch to DOR regimen, *n* (%)		12 (37.5%)
Switch to DRV/c regimen, *n* (%)		20 (62.5%)

**Table 2:** Scores before and after switch to DRV/c or DOR (*n* = 32).

Score	Pre-Switch	4 Weeks	12 Weeks	*p*-Value
PHQ-9, median (IQR), points	19 (15–22)	7 (5–12)	5 (2–12)	0.004
ISI, median, (IQR), points	19 (17–21)	11 (5–15)	7 (4–12)	0.001
HADS-A, median, (IQR), points	12 (11–194)	8 (5–11)	7 (6–9)	0.017
HADS-D, (IQR), median, points	9 (7–12)	9 (7–10)	8 (3–10)	0.001
PSQI median, (IQR) points	14 (12–15)	7 (5–8)	6 (6–7)	0.035

## Data Availability

The original contributions presented in the study are included in the article/Supplementary Materials; further inquiries can be directed to the corresponding author.

## Supplementary Materials

The following supporting information can be downloaded at: https://www.mdpi.com/article/10.3390/v16071083/s1, Supplementary S1: Screening tools for neuropsychiatric adverse events; Supplementary S2: Interpretation of neuropsychiatric scales.

## References

[1] Kunisaki K.M., De Francesco D., Sabin C.A., Winston A., Mallon P.W.G., Anderson J., Bagkeris E., Boffito M., Doyle N., Haddow L. (2020). Sleep Disorders in Human Immunodeficiency Virus: A Substudy of the Pharmacokinetics and Clinical Observations in People Over Fifty (POPPY) Study. Open Forum Infect. Dis..

[2] Abdu Z., Dule A. (2020). Poor Quality of Sleep Among HIV-Positive Persons in Ethiopia. HIV AIDS.

[3] Mazzitelli M., Trunfio M., Milinkovic A., Castelli E., Sasset L., Leoni D., Salvucci M., Cazzaro R., Calcinoni I., Balducci P. (2023). Sleep disturbances and their correlation with cardiovascular risk, obesity, and mood disorders in people with HIV. AIDS.

[4] de Boer M.G., van den Berk G.E., van Holten N., Oryszcyn J.E., Dorama W., Moha D.A., Brinkman K. (2016). Intolerance of dolutegravir-containing combination antiretroviral therapy regimens in real-life clinical practice. AIDS.

[5] Cuzin L., Pugliese P., Katlama C., Bani-Sadr F., Ferry T., Rey D., Lourenco J., Bregigeon S., Allavena C., Reynes J. (2019). Integrase strand transfer inhibitors and neuropsychiatric adverse events in a large prospective cohort. J. Antimicrob. Chemother..

[6] Hoffmann C., Schewe K., Fenske S., Buhk T., Sabranski M., Adam A., Hansen S., Stellbrink H.J. (2020). Short-Term Neuropsychiatric Tolerability of Bictegravir Combined with Emtricitabine/Tenofovir Alafenamide in Clinical Practice. Antivir. Ther..

[7] Camara A., Sow M.S., Touré A., Sako F.B., Camara I., Soumaoro K., Delamou A., Doukouré M. (2020). Anxiety and depression among HIV patients of the infectious disease department of Conakry University Hospital in 2018. Epidemiol. Infect..

[8] Savard J., Laberge B., Gauthier J.G., Bergeron M.G. (1999). Screening Clinical Depression in HIV-Seropositive Patients Using the Hospital Anxiety and Depression Scale. AIDS Behav..

[9] Cholera R., Gaynes B.N., Pence B.W., Bassett J., Qangule N., Macphail C., Bernhardt S., Pettifor A., Miller W.C. (2014). Validity of the Patient Health Questionnaire-9 to screen for depression in a high-HIV burden primary healthcare clinic in Johannesburg, South Africa. J. Affect. Disord..

[10] Milinkovic A., Singh S., Simmons B., Pozniak A., Boffito M., Nwokolo N. (2020). Multimodality assessment of sleep outcomes in people living with HIV performed using validated sleep questionnaires. Int. J. STD AIDS.

[11] Rubinstein M.L., Selwyn P.A. (1998). High prevalence of insomnia in an outpatient population with HIV infection. J. Acquir. Immune Defic. Syndr. Hum. Retrovirol..

[12] Cabello-Úbeda A., Baeza A.G., García J.T., de La Fuente Moral S., Mena M.N., Martínez A.P., Micán R., Górgolas M., Tascón G.C., de Santiago A.D. (2022). Changes in Quality of Sleep, Mood, and Other Neuropsychiatric Symptoms After Switching Dolutegravir/Lamivudine/Abacavir to Darunavir/Cobicistat/Emtricitabine/Tenofovir Alafenamide in a Randomized Study of People With Human Immunodeficiency Virus With Poor Sleep Quality: GESIDA 10418. Open Forum Infect. Dis..

[13] Taramasso L., Orofino G., Ricci E., Menzaghi B., De Socio G.V., Squillace N., Madeddu G., Vichi F., Celesia B.M., Molteni C. (2022). Reversibility of Central Nervous System Adverse Events in Course of Art. Viruses.

[14] Cipriani A., Brambilla P., Furukawa T., Geddes J., Gregis M., Hotopf M., Malvini L., Barbui C. (2005). Fluoxetine versus other types of pharmacotherapy for depression. Cochrane Database Syst. Rev..

[15] Machado-Vieira R., Baumann J., Wheeler-Castillo C., Latov D., Henter I.D., Salvadore G., Zarate C.A. (2010). The Timing of Antidepressant Effects: A Comparison of Diverse Pharmacological and Somatic Treatments. Pharmaceuticals.

[16] National Health Service (NHS) United Kigndom (2022). NHS Data [Internet]. https://www.nhs.uk/medicines/sertraline/about-sertraline/.

[17] Nierenberg A.A., Farabaugh A.H., Alpert J.E., Gordon J., Worthington J.J., Rosenbaum J.F., Fava M. (2000). Timing of onset of antidepressant response with fluoxetine treatment. Am. J. Psychiatry.

[18] Capetti A.F., Di Giambenedetto S., Latini A., Sterrantino G., De Benedetto I., Cossu M.V., Gori A. (2018). Morning dosing for dolutegravir-related insomnia and sleep disorders. HIV Med..

[19] Deike L.G., Barreiro P., Reneses B. (2023). The new profile of psychiatric disorders in patients with HIV infection. AIDS Rev..

[20] Gallego L., Barreiro P., del Río R., González de Requena D., Rodríguez-Albariño A., González-Lahoz J., Soriano V. (2004). Analyzing sleep abnormalities in HIV-infected patients treated with Efavirenz. Clin. Infect. Dis..

[21] Shikuma C.M., Kohorn L., Paul R., Chow D.C., Kallianpur K.J., Walker M., Souza S., Gangcuangco L.M.A., Nakamoto B.K., Pien F.D. (2018). Sleep and neuropsychological performance in HIV+ subjects on efavirenz-based therapy and response to switch in therapy. HIV Clin. Trials.

[22] Petrakis V., Steiropoulos P., Papanas N., Trypsianis G., Papazoglou D., Panagopoulos P. (2023). Quality of sleep in people living with HIV in the era of highly active antiretroviral treatment. Int. J. STD AIDS.

[23] O’Brien K.E., Riddell N.E., Gómez-Olivé F.X., Rae D.E., Scheuermaier K., von Schantz M. (2022). Sleep disturbances in HIV infection and their biological basis. Sleep Med. Rev..

[24] Bruno G., Giotta M., Perelli S., Spada V., Purgatorio M.A., Bartolomeo N., Buccoliero G.B. (2023). Prevalence and Risk Factors for Poor Sleep Quality in People Living with HIV: Preliminary Observations from an HIV Outpatient Clinic. Viruses.

[25] Capeau J., Lagathu C., Béréziat V. (2024). Recent data on the role of antiretroviral therapy in weight gain and obesity in persons living with HIV. Curr. Opin. HIV AIDS.

[26] Chen C.C., Liu H.Y., Chen Y.C., Ko N.Y. (2022). Relationships Among Trajectories of Sleep Disturbance, Depression, and Antiretroviral Therapy in Persons Newly Diagnosed with HIV: A One-and-a-Half-Year Observational Longitudinal Study. Nat. Sci. Sleep..

[27] Hoffmann C., Welz T., Sabranski M., Kolb M., Wolf E., Stellbrink H.J., Wyen C. (2017). Higher rates of neuropsychiatric adverse events leading to dolutegravir discontinuation in women and older patients. HIV Med..

[28] Gutierrez J., Tedaldi E.M., Armon C., Patel V., Hart R., Buchacz K. (2019). Sleep disturbances in HIV-infected patients associated with depression and high risk of obstructive sleep apnea. SAGE Open Med..

[29] Tan T., Zhou C., Lu R., Chen C., Bai C., Li L., Wu G. (2022). Depression and Associated Factors Among Men Living with HIV/AIDS Aged 50 Years and Over in Chongqing, China. J. Multidiscip. Healthc..

[30] Waters L., Winston A., Reeves I., Boffito M., Churchill D., Cromarty B., Dunn D., Fink D., Fidler S., Foster C. (2022). BHIVA guidelines on antiretroviral treatment for adults living with HIV-1 2022. HIV Med..

[31] Horne R., Chapman S., Glendinning E., Date H.L., Guitart J., Cooper V. (2019). Mind Matters: Treatment Concerns Predict the Emergence of Antiretroviral Therapy Side Effects in People with HIV. AIDS Behav..

