# Supplementary material for: Reversibility of Neuropsychiatric Adverse Events after Switching to Darunavir/Cobicistat or Doravirine in Men on INSTI-Based Regimen
Source: Viruses. 2024 Jul 5;16(7):1083. doi: 10.3390/v16071083 (PMC11281698; doi:10.3390/v16071083)
Supplement: Supplementary file 1 [file viruses-16-01083-s001.zip › viruses-2958175-supplementary.pdf]

Supplementary Materials

**1. Screening tools for neuropsychiatric adverse events.**

## Hospital Anxiety and Depression Scale (HADS)

**Tick the box beside the reply that is closest to how you have been feeling in the past week.**
**Don't take too long over you replies: your immediate is best.**

| D | A | | D | A | |
|---|---|---|---|---|---|
| | | **I feel tense or 'wound up':** | | | **I feel as if I am slowed down:** |
| | 3 | Most of the time | 3 | | Nearly all the time |
| | 2 | A lot of the time | 2 | | Very often |
| | 1 | From time to time, occasionally | 1 | | Sometimes |
| | 0 | Not at all | 0 | | Not at all |
| | | | | | |
| | | **I still enjoy the things I used to enjoy:** | | | **I get a sort of frightened feeling like 'butterflies' in the stomach:** |
| 0 | | Definitely as much | | 0 | Not at all |
| 1 | | Not quite so much | | 1 | Occasionally |
| 2 | | Only a little | | 2 | Quite Often |
| 3 | | Hardly at all | | 3 | Very Often |
| | | | | | |
| | | **I get a sort of frightened feeling as if something awful is about to happen:** | | | **I have lost interest in my appearance:** |
| | 3 | Very definitely and quite badly | 3 | | Definitely |
| | 2 | Yes, but not too badly | 2 | | I don't take as much care as I should |
| | 1 | A little, but it doesn't worry me | 1 | | I may not take quite as much care |
| | 0 | Not at all | 0 | | I take just as much care as ever |
| | | | | | |
| | | **I can laugh and see the funny side of things:** | | | **I feel restless as I have to be on the move:** |
| 0 | | As much as I always could | | 3 | Very much indeed |
| 1 | | Not quite so much now | | 2 | Quite a lot |
| 2 | | Definitely not so much now | | 1 | Not very much |
| 3 | | Not at all | | 0 | Not at all |
| | | **Worrying thoughts go through my mind:** | | | **I look forward with enjoyment to things:** |
| | 3 | A great deal of the time | 0 | | As much as I ever did |
| | 2 | A lot of the time | 1 | | Rather less than I used to |
| | 1 | From time to time, but not too often | 2 | | Definitely less than I used to |
| | 0 | Only occasionally | 3 | | Hardly at all |
| | | | | | |
| | | **I feel cheerful:** | | | **I get sudden feelings of panic:** |
| 3 | | Not at all | | 3 | Very often indeed |
| 2 | | Not often | | 2 | Quite often |
| 1 | | Sometimes | | 1 | Not very often |
| 0 | | Most of the time | | 0 | Not at all |
| | | | | | |
| | | **I can sit at ease and feel relaxed:** | | | **I can enjoy a good book or radio or TV program:** |
| | 0 | Definitely | 0 | | Often |
| | 1 | Usually | 1 | | Sometimes |
| | 2 | Not Often | 2 | | Not often |
| | 3 | Not at all | 3 | | Very seldom |

Please check you have answered all the questions

Scoring:
Total score: Depression (D) ___________ Anxiety (A) ______________
0-7 = Normal
8-10 = Borderline abnormal (borderline case)
11-21 = Abnormal (case)

## PATIENT HEALTH QUESTIONNAIRE (PHQ-9)

**ID #:** ______________________________ **DATE:**__________________

| Over the last *2 weeks,* how often have you been bothered by any of the following problems? *(use "✓" to indicate your answer)* | **Not at all** | **Several days** | **More than half the days** | **Nearly every day** |
|---|---|---|---|---|
| **1.** Little interest or pleasure in doing things | 0 | 1 | 2 | 3 |
| **2.** Feeling down, depressed, or hopeless | 0 | 1 | 2 | 3 |
| **3.** Trouble falling or staying asleep, or sleeping too much | 0 | 1 | 2 | 3 |
| **4.** Feeling tired or having little energy | 0 | 1 | 2 | 3 |
| **5.** Poor appetite or overeating | 0 | 1 | 2 | 3 |
| **6.** Feeling bad about yourself — or that you are a failure or have let yourself or your family down | 0 | 1 | 2 | 3 |
| **7.** Trouble concentrating on things, such as reading the newspaper or watching television | 0 | 1 | 2 | 3 |
| **8.** Moving or speaking so slowly that other people could have noticed. Or the opposite — being so figety or restless that you have been moving around a lot more than usual | 0 | 1 | 2 | 3 |
| **9.** Thoughts that you would be better off dead, or of hurting yourself | 0 | 1 | 2 | 3 |
| | add columns | | + | + |
| *(Healthcare professional: For interpretation of TOTAL, please refer to accompanying scoring card).* | TOTAL: | | | |

**10.** If you checked off *any problems,* how *difficult* have these problems made it for you to do your work, take care of things at home, or get along with other people?

- Not difficult at all ________
- Somewhat difficult ________
- Very difficult ________
- Extremely difficult ________

## Insomnia Severity Index (ISI)

**Name:** ______________________________ **Date:** ________________________

1. Please rate the current (i.e., last 2 weeks) **SEVERITY** of your insomnia problem(s).

| | None | Mild | Moderate | Severe | Very |
|---|---|---|---|---|---|
| Difficulty falling asleep: | 0 | 1 | 2 | 3 | 4 |
| Difficulty staying asleep: | 0 | 1 | 2 | 3 | 4 |
| Problem waking up too early: | 0 | 1 | 2 | 3 | 4 |

2. How **SATISFIED**/dissatisfied are you with your current sleep pattern?

| Very Satisfied | | | | Very Dissatisfied |
|---|---|---|---|---|
| 0 | 1 | 2 | 3 | 4 |

3. To what extent do you consider your sleep problem to **INTERFERE** with your daily functioning (e.g. daytime fatigue, ability to function at work/daily chores, concentration, memory, mood, etc.).

| Not at all Interfering | A Little | Somewhat | Much | Very Much Interfering |
|---|---|---|---|---|
| 0 | 1 | 2 | 3 | 4 |

4. How **NOTICEABLE** to others do you think your sleeping problem is in terms of impairing the quality of your life?

| Not at all Noticeable | Barely | Somewhat | Much | Very Much Noticeable |
|---|---|---|---|---|
| 0 | 1 | 2 | 3 | 4 |

5. How **WORRIED**/distressed are you about your current sleep problem?

| Not at all | A Little | Somewhat | Much | Very Much |
|---|---|---|---|---|
| 0 | 1 | 2 | 3 | 4 |

**Guidelines for Scoring/Interpretation:**

Add scores for all seven items (1a+1b+1c+ 2+3+4+5) = _____
Total score ranges from 0-28
0-7 = No clinically significant insomnia
8-14 = Subthreshold insomnia
15-21 = Clinical insomnia (moderate severity)
22-28 = Clinical insomnia (severe)

Subject's Initials________________ID#__________________________Date_________________Time_____AM PM

**PITTSBURGH SLEEP QUALITY INDEX**

**INSTRUCTIONS:**
The following questions relate to your usual sleep habits during the past month only. Your answers should indicate the most accurate reply for the majority of days and nights in the past month. Please answer all questions.

1. During the past month, what time have you usually gone to bed at night?

   BED TIME ___________

2. During the past month, how long (in minutes) has it usually taken you to fall asleep each night?

   NUMBER OF MINUTES ___________

3. During the past month, what time have you usually gotten up in the morning?

   GETTING UP TIME ___________

4. During the past month, how many hours of actual sleep did you get at night? (This may be different than the number of hours you spent in bed.)

   HOURS OF SLEEP PER NIGHT ___________

***For each of the remaining questions, check the one best response. Please answer all questions.***

5. During the past month, how often have you had trouble sleeping because you . . .

a) Cannot get to sleep within 30 minutes

| Not during the past month_____ | Less than once a week_____ | Once or twice a week_____ | Three or more times a week_____ |
|---|---|---|---|

b) Wake up in the middle of the night or early morning

| Not during the past month_____ | Less than once a week_____ | Once or twice a week_____ | Three or more times a week_____ |
|---|---|---|---|

c) Have to get up to use the bathroom

| Not during the past month_____ | Less than once a week_____ | Once or twice a week_____ | Three or more times a week_____ |
|---|---|---|---|

d) Cannot breathe comfortably

| Not during the past month_____ | Less than once a week_____ | Once or twice a week_____ | Three or more times a week_____ |
|---|---|---|---|

e) Cough or snore loudly

| Not during the past month_____ | Less than once a week_____ | Once or twice a week_____ | Three or more times a week_____ |
|---|---|---|---|

f) Feel too cold

| Not during the past month_____ | Less than once a week_____ | Once or twice a week_____ | Three or more times a week_____ |
|---|---|---|---|

g) Feel too hot

| Not during the past month_____ | Less than once a week_____ | Once or twice a week_____ | Three or more times a week_____ |
|---|---|---|---|

h) Had bad dreams

| Not during the past month_____ | Less than once a week_____ | Once or twice a week_____ | Three or more times a week_____ |
|---|---|---|---|

i) Have pain

| Not during the past month_____ | Less than once a week_____ | Once or twice a week_____ | Three or more times a week_____ |
|---|---|---|---|

j) Other reason(s), please describe_______________________________________________

_____________________________________________________________________________

How often during the past month have you had trouble sleeping because of this?

| Not during the past month_____ | Less than once a week_____ | Once or twice a week_____ | Three or more times a week_____ |
|---|---|---|---|

6. During the past month, how would you rate your sleep quality overall?

Very good ____________

Fairly good ____________

Fairly bad ____________

Very bad ____________

7. During the past month, how often have you taken medicine to help you sleep (prescribed or "over the counter")?

Not during the past month_____ Less than once a week_____ Once or twice a week_____ Three or more times a week_____

8. During the past month, how often have you had trouble staying awake while driving, eating meals, or engaging in social activity?

Not during the past month_____ Less than once a week_____ Once or twice a week_____ Three or more times a week_____

9. During the past month, how much of a problem has it been for you to keep up enough enthusiasm to get things done?

No problem at all __________

Only a very slight problem __________

Somewhat of a problem __________

A very big problem __________

10. Do you have a bed partner or room mate?

No bed partner or room mate __________

Partner/room mate in other room __________

Partner in same room, but not same bed __________

Partner in same bed __________

If you have a room mate or bed partner, ask him/her how often in the past month you have had . . .

a) Loud snoring

Not during the past month_____ Less than once a week_____ Once or twice a week_____ Three or more times a week_____

b) Long pauses between breaths while asleep

Not during the past month_____ Less than once a week_____ Once or twice a week_____ Three or more times a week_____

c) Legs twitching or jerking while you sleep

Not during the past month_____ Less than once a week_____ Once or twice a week_____ Three or more times a week_____

d) Episodes of disorientation or confusion during sleep

Not during the past month_____ Less than once a week_____ Once or twice a week_____ Three or more times a week_____

e) Other restlessness while you sleep; please describe________________________________________________

________________________________________________________________________________

Not during the past month_____ Less than once a week_____ Once or twice a week_____ Three or more times a week_____

**2. Interpretation of neuropsychiatric scales.**

| | |
|---|---|
| **HADS-D / HADS-A** | |
| 0-7 points | Normal |
| 8-10 points | Borderline case |
| 11-21 points | Abnormal (case) |
| **PHQ-9** | |
| 1-4 points | Minimal depression |
| 5-9 points | Mild depression |
| 10-14 points | Moderate depression |
| 15-19 points | Moderately severe depression |
| 20-27 points | Severe depression |
| **ISI** | |
| 0-7 points | No clinically significant insomnia |
| 8-14 points | Subthreshold insomnia |
| 15-21 points | Clinical insomnia (moderate severity) |
| 22-28 points | Clinical insomnia (severe) |
| **PSQI** | |
| ≤ 5 | Associated with good sleep quality |
| > 5 | Associated with poor sleep quality |
